# Supplementary material for: Mechanisms governing the pioneering and redistribution capabilities of the non-classical pioneer PU.1
Source: Nat Commun. 2020 Jan 21;11:402. doi: 10.1038/s41467-019-13960-2 (PMC6972792; doi:10.1038/s41467-019-13960-2)
Supplement: Supplementary file 7 — Source data [file 41467_2019_13960_MOESM7_ESM.zip › Source_Data/FigureS2/FigureS2B_MotifScanOutput/KmeansCluster12/homerResults/motif21.similar.html]

motif21

## Information for motif21

G
A
T
C
G
A
T
C
G
T
C
A
T
A
G
C
A
G
C
T
A
G
C
T
A
G
T
C
C
G
A
T
C
A
T
G
A
G
T
C
G
A
C
T
C
A
G
T
A
C
G
T
  
Reverse Opposite:  

T
G
C
A
G
T
C
A
C
T
G
A
T
C
A
G
G
T
A
C
C
G
T
A
C
T
A
G
T
C
G
A
T
C
G
A
A
T
C
G
C
A
G
T
C
T
A
G
C
T
A
G
  

|  |  |
| --- | --- |
| p-value: | 1e-21 |
| log p-value: | -5.018e+01 |
| Information Content per bp: | 1.725 |
| Number of Target Sequences with motif | 62.0 |
| Percentage of Target Sequences with motif | 3.45% |
| Number of Background Sequences with motif | 316.8 |
| Percentage of Background Sequences with motif | 0.69% |
| Average Position of motif in Targets | 102.4 +/- 46.4bp |
| Average Position of motif in Background | 102.6 +/- 70.3bp |
| Strand Bias (log2 ratio + to - strand density) | 0.2 |
| Multiplicity (# of sites on avg that occur together) | 1.02 |
| Motif File: | file (matrix) reverse opposite |

### Similar de novo motifs found

|  |  |  |  |  |  |  |  |
| --- | --- | --- | --- | --- | --- | --- | --- |
| Rank | Match Score | Redundant Motif | P-value | log P-value | % of Targets | % of Background | Motif file |
| 1 | 0.890 | T C A G T C G A C G T A C G T A C G A T C T A G T A G C C G T A C T A G G T C A C G T A T C A G A G C T A T C G | 1e-16 | -38.741892 | 3.06% | 0.71% | motif file (matrix) |
| 2 | 0.761 | G T C A A G T C G C T A A C G T A G T C A C G T A C T G A G T C C G T A A C G T A C G T A C G T | 1e-13 | -30.325571 | 0.78% | 0.03% | motif file (matrix) |
| 3 | 0.804 | C G T A C G T A A C G T A C T G A G T C C G T A A C T G G T C A C G T A A C T G A C G T | 1e-12 | -28.198289 | 1.11% | 0.11% | motif file (matrix) |
